# Improving the Prognostic Evaluation Precision of Hospital Outcomes for Heart Failure Using Admission Notes and Clinical Tabular Data: Multimodal Deep Learning Model

**DOI:** 10.2196/54363

**Published:** 2024-05-02

**Authors:** Zhenyue Gao, Xiaoli Liu, Yu Kang, Pan Hu, Xiu Zhang, Wei Yan, Muyang Yan, Pengming Yu, Qing Zhang, Wendong Xiao, Zhengbo Zhang

**Affiliations:** 1 Beijing Engineering Research Center of Industrial Spectrum Imaging School of Automation and Electrical Engineering University of Science and Technology Beijing Beijing China; 2 Center for Artificial Intelligence in Medicine The General Hospital of People's Liberation Army Beijing China; 3 Department of Cardiology West China Hospital Sichuan University Chengdu China

**Keywords:** heart failure, multimodal deep learning, mortality prediction, admission notes, clinical tabular data, tabular, notes, deep learning, machine learning, cardiology, heart, cardiac, documentation, prognostic, prognosis, prognoses, predict, prediction, predictions, predictive

## Abstract

**Background:**

Clinical notes contain contextualized information beyond structured data related to patients’ past and current health status.

**Objective:**

This study aimed to design a multimodal deep learning approach to improve the evaluation precision of hospital outcomes for heart failure (HF) using admission clinical notes and easily collected tabular data.

**Methods:**

Data for the development and validation of the multimodal model were retrospectively derived from 3 open-access US databases, including the Medical Information Mart for Intensive Care III v1.4 (MIMIC-III) and MIMIC-IV v1.0, collected from a teaching hospital from 2001 to 2019, and the eICU Collaborative Research Database v1.2, collected from 208 hospitals from 2014 to 2015. The study cohorts consisted of all patients with critical HF. The clinical notes, including chief complaint, history of present illness, physical examination, medical history, and admission medication, as well as clinical variables recorded in electronic health records, were analyzed. We developed a deep learning mortality prediction model for in-hospital patients, which underwent complete internal, prospective, and external evaluation. The Integrated Gradients and SHapley Additive exPlanations (SHAP) methods were used to analyze the importance of risk factors.

**Results:**

The study included 9989 (16.4%) patients in the development set, 2497 (14.1%) patients in the internal validation set, 1896 (18.3%) in the prospective validation set, and 7432 (15%) patients in the external validation set. The area under the receiver operating characteristic curve of the models was 0.838 (95% CI 0.827-0.851), 0.849 (95% CI 0.841-0.856), and 0.767 (95% CI 0.762-0.772), for the internal, prospective, and external validation sets, respectively. The area under the receiver operating characteristic curve of the multimodal model outperformed that of the unimodal models in all test sets, and tabular data contributed to higher discrimination. The medical history and physical examination were more useful than other factors in early assessments.

**Conclusions:**

The multimodal deep learning model for combining admission notes and clinical tabular data showed promising efficacy as a potentially novel method in evaluating the risk of mortality in patients with HF, providing more accurate and timely decision support.

## Introduction

Heart failure (HF), a syndrome of impaired heart function, represents the advanced stage of various cardiac conditions [1-3]. With its substantial influence on both morbidity and mortality, HF poses a formidable challenge to human health and societal progress [4-6].

As a potentially life-threatening condition, particularly when accompanied by advanced organ dysfunction or severe complications, a considerable portion of patients with HF may require immediate access to advanced, high-technology, life-saving care, which is typically available only in intensive care units (ICUs) [7]. Studies have indicated that approximately 10% to 51% of patients with HF admitted to hospitals in the United States are subsequently admitted to ICUs [8,9]. It has also been found that ICU-admitted patients with HF experience significantly higher adjusted in-hospital mortality rates compared to those admitted solely to hospitals [10]. The in-hospital mortality rate for patients with HF receiving treatment in an ICU has been reported as 10.6%, in contrast to the overall in-hospital mortality rate of 4% for all patients with HF [11]. Given this substantially higher mortality rate, accurate prediction of in-hospital mortality could empower physicians to implement early interventions and tailor individualized treatments [12,13]. Consequently, there is an increasing need for the development of predictive models that can effectively identify individuals at a heightened risk of mortality in the ICU.

Most previous research works have applied statistical analysis or machine learning techniques using structured administrative data from electronic health records to identify significant risk predictors that trigger adverse outcomes [14-19]. However, HF disease often develops rapidly, and while some sensitive biomarkers, such as N-terminal pro–b-type natriuretic peptide (NT-proBNP), tend to increase in reactivity after the disease progresses, their efficiency is limited due to their high cost and inability to be measured in real time [20,21]. Recently, there has been a growing acknowledgment of the importance of clinical narratives in clinical decision-making [22,23]. The narrative notes at admission, such as chief complaint, history of present illness, physical examination, medical history, and admission medication, play a central role in health care communication. They represent a more comprehensive and personalized account of patient history and assessments [24]. Harnessing the potential of clinical narratives can largely enhance patient care and contribute to the improvement of predictive models for prognosis [25,26]. Exploiting the potential of clinical narratives and modeling them by multimodal deep learning (DL) approaches can enhance the precision of patient care and contribute to the improvement of predictive models for in-hospital mortality.

We aim to design a multimodal DL model and explore the infusion approaches to improve evaluation performance using tabular data and admission notes. The cross-modal model, which characterizes textual, categorical, and continuous variables separately, significantly outperforms the unimodal models on the multicenter and prospective validation sets. We believe our findings will motivate data-centric studies to more precisely characterize the illness severity of patients with HF.

## Methods

### Study Design

**Figure 1:**
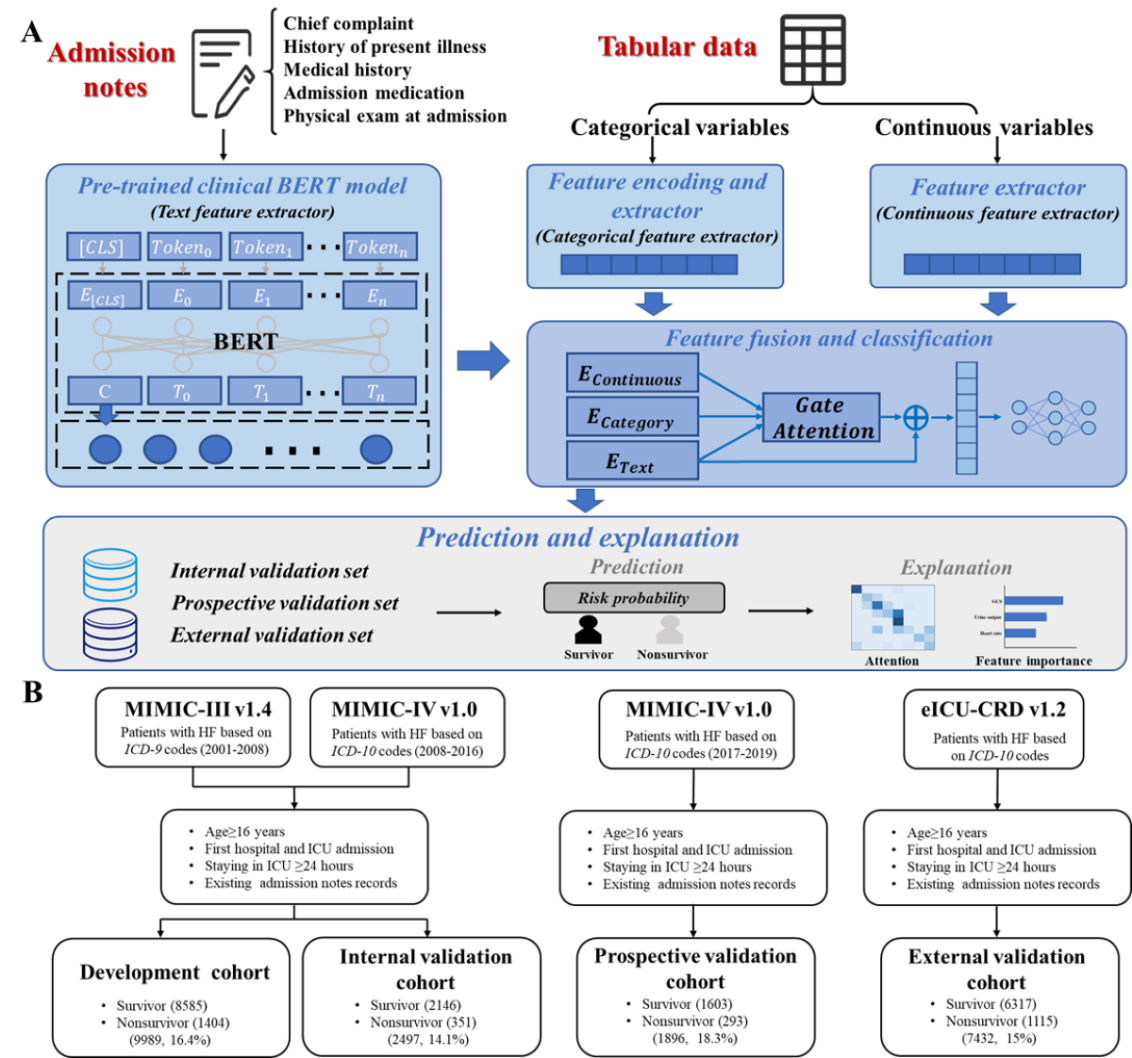
(A) Workflow of the multimodal deep learning model with the fusion of textual and tabular data. (B) An overview of the inclusion criteria with all study cohorts. BERT: Bidirectional Encoder Representations from Transformers; eICU-CRD: eICU Collaborative Research Database; HF: heart failure; *ICD-10: International Classification of Diseases, Tenth Revision*; ICU: intensive care unit; MIMIC: Medical Information Mart for Intensive Care.

An overview of the study flow is shown in Figure 1A. First, we acquired patients’ admission notes and tabular data, and these two single modalities were separately embedded to obtain the feature and status representation. The categorical and continuous variables in tabular data were characterized separately. Next, a feature-fusing DL network was applied to integrate the two modalities and achieved the model development. Then, a fully connected DL network was used to predict the in-hospital outcome, our primary outcome of interest. Two postexplanation approaches were adopted to increase the credibility of the model. Finally, the internal, prospective, and external validation with multiple evaluation metrics were accomplished. Our study followed the Transparent Reporting of a Multivariable Prediction Model for Individual Prognosis or Diagnosis (TRIPOD) reporting guidelines for prognosis studies.

### Data Sets and Cohorts

The cohorts for this multicenter retrospective cohort study were derived from 3 open-access clinical databases, including the Medical Information Mart for Intensive Care v1.4 (MIMIC-III; CareVue) and MIMIC-IV v1.0, collected from the Beth Israel Deaconess Medical Center in Boston from 2001 to 2008 and 2008 to 2019, respectively [27,28], and the eICU Collaborative Research Database v1.2 (eICU-CRD), collected from 208 hospitals in United States from 2014 to 2015 [29]. We included all first-time ICU admissions for patients with HF aged ≥16 years according to the International Classification of Diseases diagnostic codes. We excluded patients who stayed in the ICU for less than 24 hours and did not have admission notes. Patients were divided into 4 cohorts to support adequate model evaluation, including the development, internal validation, prospective validation, and external validation cohorts. These cohorts correspond to different stages of model development and evaluation. The development cohort consisted of a subset of data used to create or develop the predictive model. Internal validation, prospective validation, and external validation cohorts helped to check if the model had learned patterns that generalized well to new, unseen data. Each of these cohorts played a crucial role in different stages of model development and validation, ensuring that the predictive model was accurate, reliable, and applicable to new and diverse data sets or situations. The inclusion criteria of them are displayed in Figure 1B.

### Data Extraction

Our target is to provide early clinical decision support during ICU admissions. The 5 types of commonly recorded notes were extracted, including chief complaint, history of present illness, medical history, admission medications, and physical exam. In Figure S1 in Multimedia Appendix 1, an example of an admission note is shown with highlights. Meanwhile, 6 types of clinical variables were collected for model development, as follows: (1) basic information of age, gender, weight, BMI, and Charlson Comorbidity Index; (2) vital signs, such as Glasgow Coma Scale, heart rate, respiratory rate, and systolic blood pressure; (3) laboratory tests, including glucose, creatinine, white blood cell, and total bilirubin; (4) urine output; (5) treatments received, including mechanical ventilation; and (6) physical frailty assessments, including activity and fall risks. Representative statistical features were calculated based on the type of variable, such as the maximum, minimum, and mean values. The median value of each feature was used to impute missing values for continuous variables except for FiO_2_ ([fraction of inspired oxygen] with the imputation of 21%), with a missing ratio limitation of less than 30%. Details about all types of candidate variables are provided in Table S1 in Multimedia Appendix 1. Their missing ratio is shown in Table S2 in Multimedia Appendix 1.

### Model Development and Output

The model was constructed based on a supervised multimodal DL framework, which mainly included feature extractors and a feature fusion module. A pretrained Bidirectional Encoder Representations from Transformers (BERT) module was used for learning the presentation of clinical notes [30]. In the preliminary experiments, we used all the text chunks (the same subset from the training set for the model) to compare the performances of different pretrained clinical BERT models. We found that clinical BERT [31] demonstrated the best comprehensive performance (Table S3 in Multimedia Appendix 1). In the fusion module, a gate attention mechanism [32] was introduced to aggregate the embedded features of clinical notes and tabular data using attention scores; this module finally output the predicted risk probability of in-hospital deaths through a fully connected layer (Figure S2 in Multimedia Appendix 1). The maximum predicted value of all text chunks for a patient was adopted as the optimal risk prediction score. Further detailed information on model building and training is present in the Multimedia Appendix 1.

### Model Explanation

In the pursuit of explicating the underlying mechanisms of the DL model and facilitating a comprehensive visualization of pivotal insights, we embarked on an intricate analysis of the pivotal terminologies instrumental in shaping predictions within the developed model. To achieve this, we used the Integrated Gradients (IG) technique [33] to enhance our comprehension of the BERT model’s inner workings and the rationale behind its predictions. This technique hinges on computing gradients with respect to input features, gauging each feature’s contribution to the model’s prediction. IG offers an intuitive understanding of model predictions by quantifying different features’ contributions, aiding clinicians and researchers in comprehending the model’s decisions [34,35]. At the same time, IG demonstrates stability across diverse samples and model architectures, yielding consistent explanatory outcomes, crucial in the face of clinical data diversity and complexity [36]. Consequently, it is considered a reliable analytical tool, helping to assess how each word in the input sequence influences the model's predictions for our research. Simultaneously, we harnessed the SHapley Additive exPlanations (SHAP) technique to unravel the importance of clinical variables in structured tabular data. We computed Shapley values to rank the important clinical variables. Shapley values involve a game theory–based approach to explain the prediction of DL models. They measure the contribution of a given feature value to the difference between the actual prediction and the mean prediction. It is important to note that higher SHAP values signify a heightened pertinence of specific terms in influencing the model’s predictions, whereas relatively diminished SHAP values connote a less pronounced impact. The IG technique exhibits a similar pattern.

Leveraging the IG and SHAP techniques offers valuable insights into the intricate relationship between input features and prediction outcomes, contributing to a more comprehensive understanding of the model’s decision-making process.

### Model Validation

The discrimination performance of our prediction model was assessed on the internal (MIMIC, 2001-2016), prospective (MIMIC, 2017-2019) and external (eICU-CRD, 2014-2015) validation cohorts. This assessment compared the model against different single modalities covering notes, tabular data, and a combination of both. The importance of the 5 types of notes for outcome assessment was also examined separately. We trained 5 predictive models based on the tabular data and individual clinical notes. It should be mentioned that the chief complaint was absent in the external validation cohort. Three evaluation metrics were calculated along with their corresponding 95% CIs, the area under the receiver operating characteristic curve (AUROC), *F*_1_-score, and the area under the precision-recall curve.

### Statistics Analysis

The median (IQR) values for continuous variables are presented. The *t* test (2-tailed) or the Wilcoxon Rank Sum Test was used when appropriate to compare survivors and nonsurvivors of HF. Categorical variables were reported by total numbers and percentages. Two-sided *P* values of less than .05 were considered statistically significant.

### Ethical Considerations

This study was exempt from institutional review board approval due to the retrospective design and lack of direct patient intervention. All data from patients were retrospectively collected from the electronic health care records systems (in the form of third-party public databases or hospital health care systems), which originated from daily clinical work.

All data were de-identified before the analysis. Third-party public databases (MIMIC-IV, MIMIC-III, and eICU-CRD) were used in this study. The institutional review boards of the Massachusetts Institute of Technology (number 0403000206) and Beth Israel Deaconess Medical Center (number 2001-P-001699/14) approved the use of the database for research.

The requirement for individual patient consent was waived because the study did not impact clinical care, all protected health information was deidentified, and all available data in the databases were anonymous.

## Results

### Patient Characteristics

A total of samples from 12,486 (14.1%) patients with HF were collected from MIMIC-III and MIMIC-IV joint data sets between 2001 and 2016; they were randomly divided into a development set and an independent internal validation set. Additionally, 1896 (18.3%) patients with HF were extracted from MIMIC-IV from 2017 to 2019 for a prospective validation set. For the external validation set, 7432 (15%) patients with HF were extracted from the eICU-CRD data set. Baseline characteristics are summarized in Table 1. The proportion of patients with in-hospital mortality in the 4 cohorts ranges from 14% to 19%. Detailed comparisons of survivors and nonsurvivors in all study cohorts are shown in Tables S4-S7 in Multimedia Appendix 1.

**Table 1:** The comparison of the total study cohorts for model development and validation.

Characteristics		Development set (n=9989)	Internal validation set (n=2497)	Prospective validation set (n=1896)	External validation set (n=7432)
**Basic information**					
	Age (years), median (IQR)	75 (65-84)	75 (64-83)	74 (64-82)	73 (62-82)
	Female sex, n (%)	4637 (46.4)	1151 (46.1)	786 (41.5)	3470 (46.7)
	BMI (kg/m^2^), median (IQR)	27.7 (24.1-32.6)	27.5 (23.8-32.6)	28.8 (24.4-33.8)	28.9 (24.4-35.0)
	CCI^a^ score, median (IQR)	7.0 (5.0-8.0)	7.0 (5.0-9.0)	7.0 (6.0-9.0)	5.0 (4.0-6.0)
Physical frailty (fall risk), n (%)		3334 (33.4)	836 (33.5)	1896 (100)	1374 (18.5)
**Activity, n (%)**					
	Bed	7347 (73.8)	1801 (72.6)	1064 (56.2)	3955 (83.5)
	Sit	1759 (17.7)	444 (17.9)	441 (23.3)	279 (5.9)
	Stand	843 (8.5)	235 (9.5)	388 (20.5)	504 (10.6)
**Notes recorded proportion, n (%)**					
	Chief complaint	8592 (86.0)	2146 (85.9)	1659 (87.5)	0 (0)
	History of present illness	9866 (98.8)	2455 (98.3)	1680 (88.6)	7186 (96.7)
	Medical history	9730 (97.4)	2427 (97.2)	1675 (88.3)	7188 (96.7)
	Admission medication	9018 (90.3)	2257 (90.4)	1650 (87.0)	4387 (59.0)
	Physical exam	9186 (92.0)	2303 (92.2)	1644 (86.7)	5104 (68.7)
**Outcome**					
	Days before ICU^b^ admission, median (IQR)	0.1 (0.0-1.1)	0.1 (0.0-1.3)	0.1 (0.0-0.9)	0.2 (0.1-0.8)
	Days of ICU admission, median (IQR)	3.0 (1.8-5.5)	3.0 (1.8-5.5)	2.9 (1.7-5.3)	2.8 (1.8-4.9)
	Days of hospital admission, median (IQR)	8.9 (5.7-14.6)	9.2 (5.8-14.7)	9.8 (6.0-15.7)	7.2 (4.3-11.9)
	Death in hospital, n (%)	1404 (16.4)	351 (14.1)	293 (18.3)	1115 (15.0)

^a^CCI: Charlson Comorbidity Index.

^b^ICU: intensive care unit.

### Model Performance Evaluation

**Figure 2:**
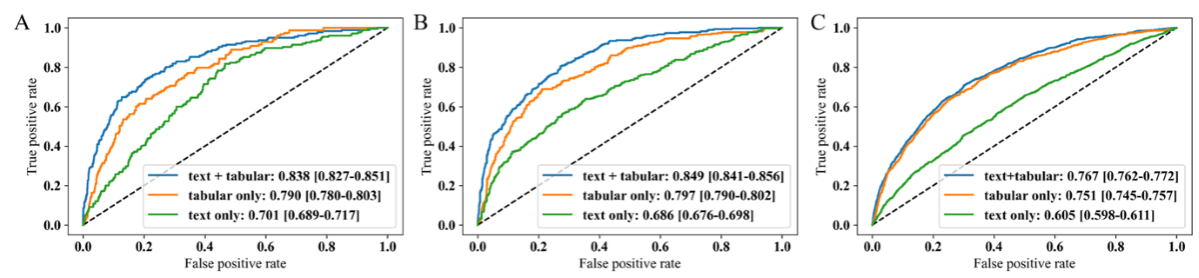
The receiver operating characteristic curve comparison of the optimal multimodal deep learning model against different single modalities in the (A) internal validation set, (B) prospective validation set, and (C) external validation set. Values inside brackets are 95% CIs.

We present the discrimination performance on internal, prospective, and external validation sets by receiver operating characteristic curves of the optimal models after tuning the hyperparameters (Figure 2). The AUROCs of the multimodal model were significantly higher than the two unimodal models in all 3 types of validation evaluations. They were 0.838 (95% CI 0.827-0.851), 0.849 (95% CI 0.841-0.856), and 0.767 [0.762-0.772] for the internal, prospective, and external validation sets, respectively. Specifically, the design details of the unimodal models were as follows: the text-based unimodal model used clinical BERT, leveraging its capabilities in contextualizing clinical text data; on the other hand, the tabular unimodal model used a fully connected model structure, tailored to effectively process structured tabular data from the tables. More comparisons on baseline models, such as random forest and logistic regression, and all evaluation metrics for these models in the 3 validation types are presented in Table S8 in Multimedia Appendix 1.

### Contribution of Individual Part in Clinical Notes

**Figure 3:**
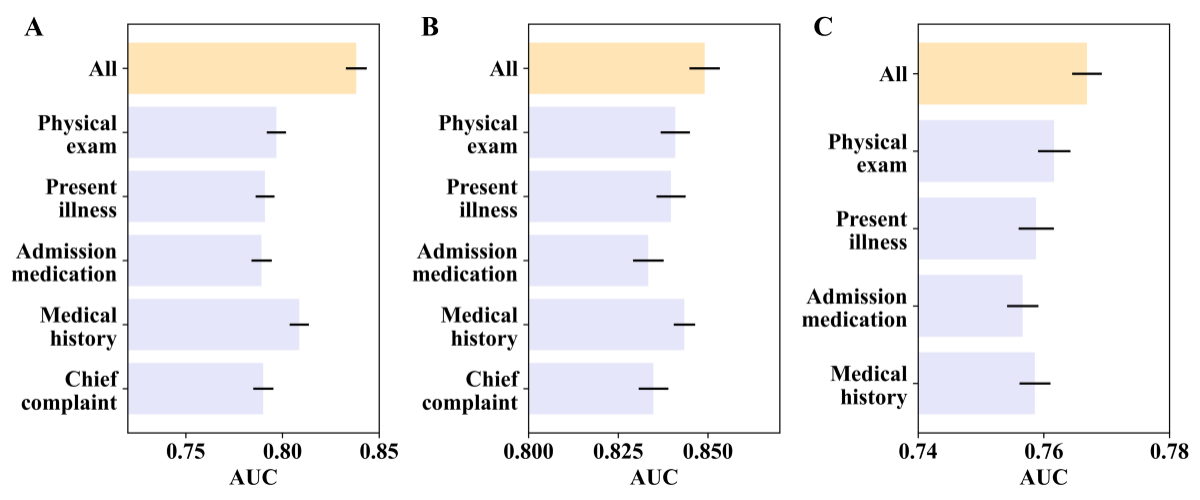
The reduction of discrimination when separately combining the individual clinical note with tabular data in the (A) internal validation set, (B) prospective validation set, and (C) external validation set. AUC: area under the receiver operating characteristic curve.

The performance contributions of the 5 types of clinical notes (including chief complaint, history of present illness, medical history, admission medication, and physical exam) were separately evaluated by combining them with clinical variables to retrain all prediction models. Figure 3 and Table S9 (Multimedia Appendix 1) display the AUROC comparisons with the full model. We found the individual contributions were much lower than the overall contribution in all validation cohorts. Specifically, medical history and physical exam contained more information that was useful in assessing the prognosis of patients with HF compared to other note types.

### Clinical Notes Visualization and Interpretation

**Figure 4:**
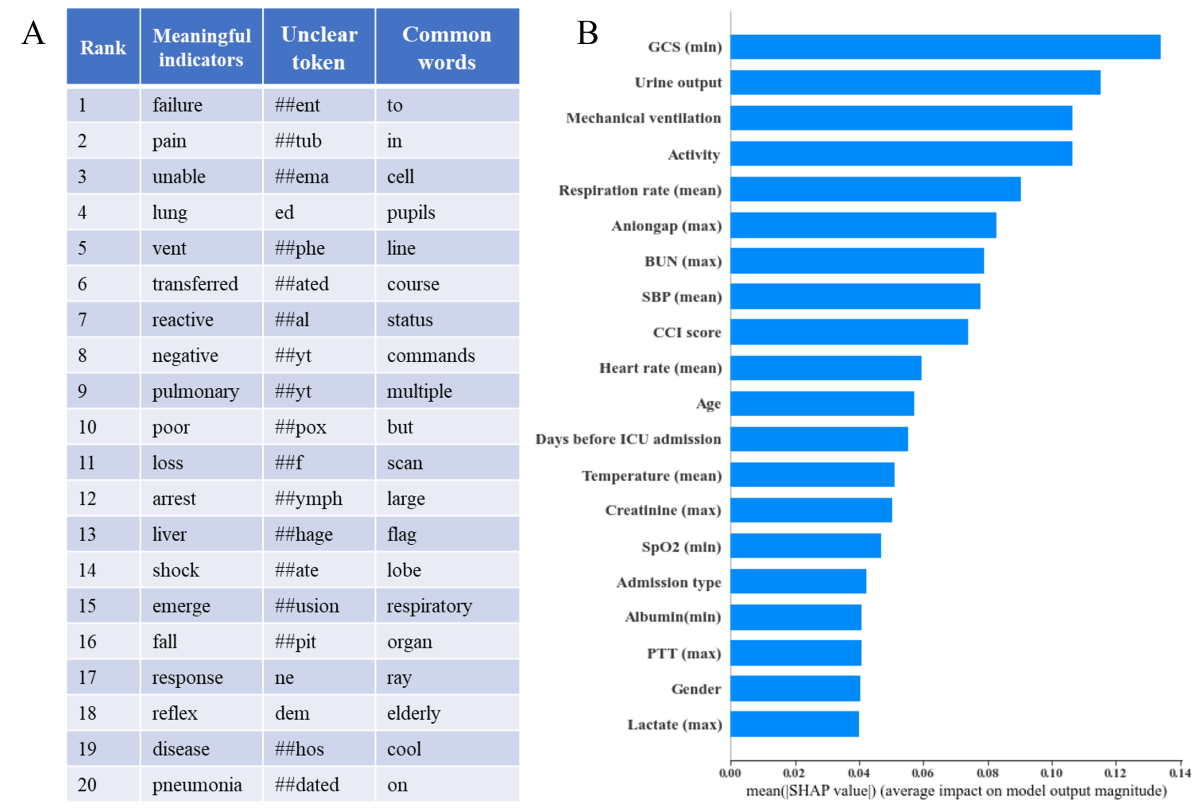
Explanation of the developed deep learning model. (A) The top 20 tokens of clinical notes evaluated by the Integrated Gradient values in 3 types of language scenarios. (B) The top 20 clinical variables evaluated by the Shapley values. BUN: blood urea nitrogen; CCI: Charlson comorbidity index; GCS: Glasgow Coma Scale; ICU: intensive care unit; PTT: partial thromboplastin time; SBP: systolic blood pressure; SHAP: SHapley Additive exPlanations; SpO_2_: oxygen saturation.

We applied the IG method to study the attribution of the prediction of a deep network to its input features, aiming to provide explanation for individual predictions. IG is computed based on the gradient of the prediction outputs with respect to the input words. Higher IG values denote the greater significance of a word to the model’s prediction, whereas lower values indicate lesser importance. We derived IG values for all tokens present in the clinical notes of each patient within the test data set, extracting those tokens with higher IG values. It is important to note that, due to BERT’s tokenization process, inputs are represented as tokens rather than individual words. For instance, the phrase “the patient has been extubated” is tokenized into “the patient has been ex ##tub ##ated” as the input sequence [35]. To enhance readability, we conducted postprocessing by excluding numbers, tokens with only 1 or 2 characters, and separators. A clinical expert assessed the clinical significance of tokens and their associated IG values in the context of mortality prediction. The sorted tokens are illustrated in Figure 4A.

The analysis identified commonly ubiquitous clinical terms like “in,” “to,” and “with,” which were segregated due to their limited potential in distinguishing prognostic variations. Among the top 20 clinically meaningful indicators vital for mortality prediction, intriguing insights emerged upon clinical interpretation. For instance, “Failure” and “Pain,” the leading predictors, denote prevalent symptoms within ICU care and can mirror disease severity and disability. Indicators 4 and 9 align with pulmonary pathology, their elevated importance reflecting the gravity of respiratory conditions and the necessity for ICU interventions, such as mechanical ventilation. Additional indicators such as “pneumonia” and “fall” manifest acute illness, carrying prognostic weight in mortality prediction. Clinical cues, such as “status,” “reflex,” and “shock,” correspond to mental well-being, with their significance in prognosis attributed to the association of delirium with adverse outcomes.

### Clinical Variables Feature Analysis

We ranked the important clinical variables using the SHAP technique. The top 20 out of 52 clinical variables (Figure 4) show that for structured tabular data, the highest ranked variables also correlate with disease severity and poorer prognosis. These variables represent clinically important information, such as mental status, using the Glasgow Coma Scale, urine output, mechanical ventilation, activity, and respiratory rate measurements.

## Discussion

### Principal Findings

This retrospective prognostic study aimed to develop, validate, and explain a multimodal DL prediction model for in-hospital outcomes in critically ill patients with HF. The model was constructed based on the admission notes and records from the first ICU admission day. Simultaneously, we compared the difference between multimodal and unimodal models and explored the individual importance of admission notes in the clinical practice of HF. We found that multimodality could further enhance the model’s ability and credibility to evaluate outcomes compared to unimodality.

Emerging clinical data sets provide an opportunity for the DL techniques to study the problem of in-hospital mortality prediction. Compared to previous related work, which mostly considers single modality or simply concatenates embeddings from different modalities, our work demonstrates a novel approach. We separately embed texts as well as categorical and continuous variables to integrate multimodal knowledge and leverage clinical notes information for better predictions. Our comprehensive experiments demonstrate that our proposed model outperforms the models using single modality (text-only AUROC: 0.701; tabular-only AUROC: 0.790) by achieving high performance (AUROC: 0.838).

The fusion method we used integrated two different modalities—unstructured clinical notes and structured clinical variables—into a universal shareable space using a transformer block. It was efficient to leverage clinical notes and integrate tabular data. Meanwhile, the novel application of an attention mechanism on clinical data enhanced the model’s ability to focus on evaluating the target in models when fusing multimodal information. Our ablation study, as shown in Figures S3-S5 (Multimedia Appendix 1), on domain adaptive pretraining and task adaptive fine-tuning with multiple BERTs verified the significance of pretraining and fine-tuning, when implementing BERT models on natural language text, especially on domain-specific clinical notes.

In the multimodal model, the proportion of clinical variables, especially continuous variables, was much higher compared to other parts (Figure S6 in Multimedia Appendix 1). The analysis and visualization of important words in clinical notes also yielded interesting findings. The ranking of words by IG values provided face validity, indicating that some of the important words used for prediction were clinically related to diseases trajectory, such as the severity of respiratory disease or mental status. Some of the unspecified words, such as “disease,” used in diverse scenarios, were more difficult to interpret as isolated words. Lastly, some of the clinically meaningful words can change significantly with negation, such as “fall.” In the future, we will use more techniques, such as the NegEx algorithm, to consider negation of keywords to better explain the clinical words’ meanings.

There are some limitations in our study. First, our model leveraged the electronic health records data based on patients’ ICU admission and the first day of admission to predict in-hospital death risk. It did not include recorded data during the treatment, which might reduce the evaluation performance of the model. Second, we simplify the feature extraction, using the maximum, minimum, or mean statistical values to characterize all data throughout the day. Such simplification ignored the changes in time series, and it might have caused the loss of useful information. Time series data will be considered in our future study. Finally, we recommend that the model need to be calibrated using local data to avoid assessment bias.

### Conclusions

In this multicenter prognostic study, we developed and validated an attention-multimodal DL model for in-hospital outcome prediction of patients with HF and explored the approaches that can improve the evaluation precision by simultaneously characterizing both admission notes and tabular data. The AUROCs of our model were significantly higher than those of unimodal models in all validation sets. The clinical variables included in the study made a particularly significant contribution to the overall results, with the data from the clinical notes exhibiting a much lower contribution. The model shows good predictive and explainable performance to potentially support the precise decision-making and disease management of critically ill patients with HF.

## Appendix

Multimedia Appendix 1 Additional statistics.

## Abbreviations

AUROC: area under the receiver operating characteristic curve
BERT: Bidirectional Encoder Representations from Transformers
DL: deep learning
eICU-CRD: eICU Collaborative Research Database
HF: heart failure
ICU: intensive care unit
IG: Integrated Gradients
MIMIC: Medical Information Mart for Intensive Care
NT-proBNP: N-terminal pro–b-type natriuretic peptide
SHAP: SHapley Additive exPlanations
