# Supplementary material for: Improving the Prognostic Evaluation Precision of Hospital Outcomes for Heart Failure Using Admission Notes and Clinical Tabular Data: Multimodal Deep Learning Model
Source: J Med Internet Res. 2024 May 2;26:e54363. doi: 10.2196/54363 (PMC11099809; doi:10.2196/54363)
Supplement: Multimedia Appendix 1 [file jmir_v26i1e54363_app1.docx]

**Appendix**

**Figure S1. An example of clinical notes at admission**

**
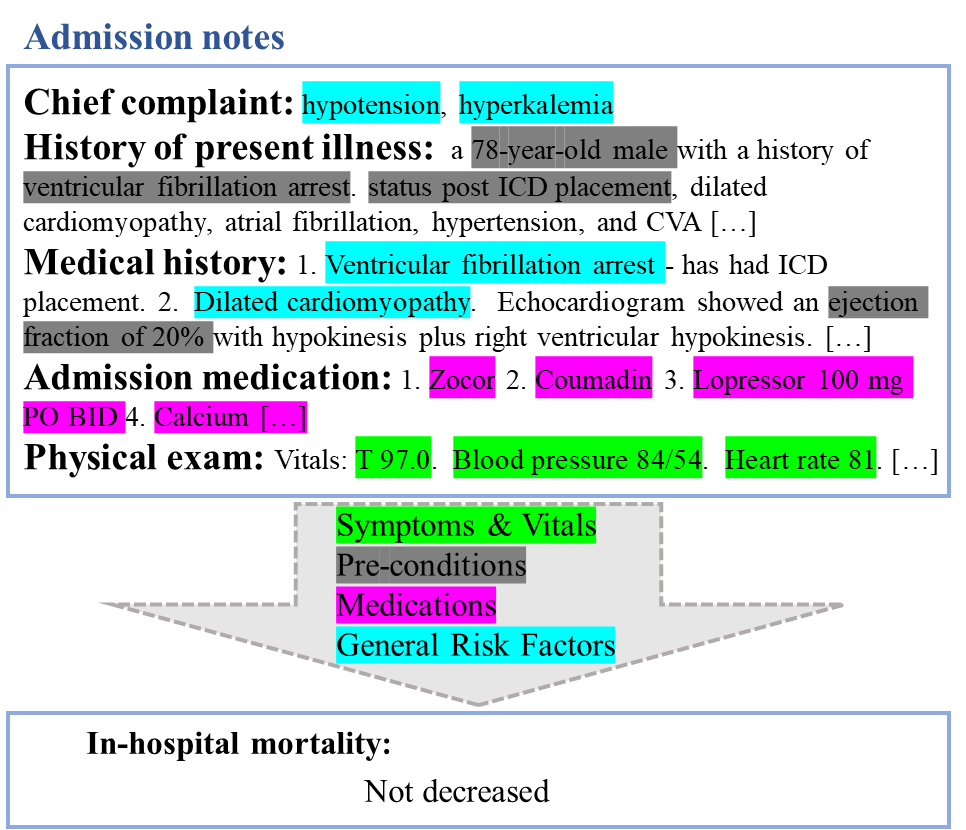
**

**
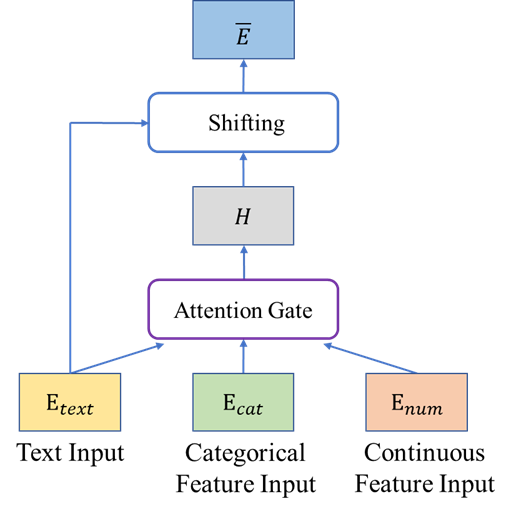
**

**Figure S2. Network architecture of feature fusion.**


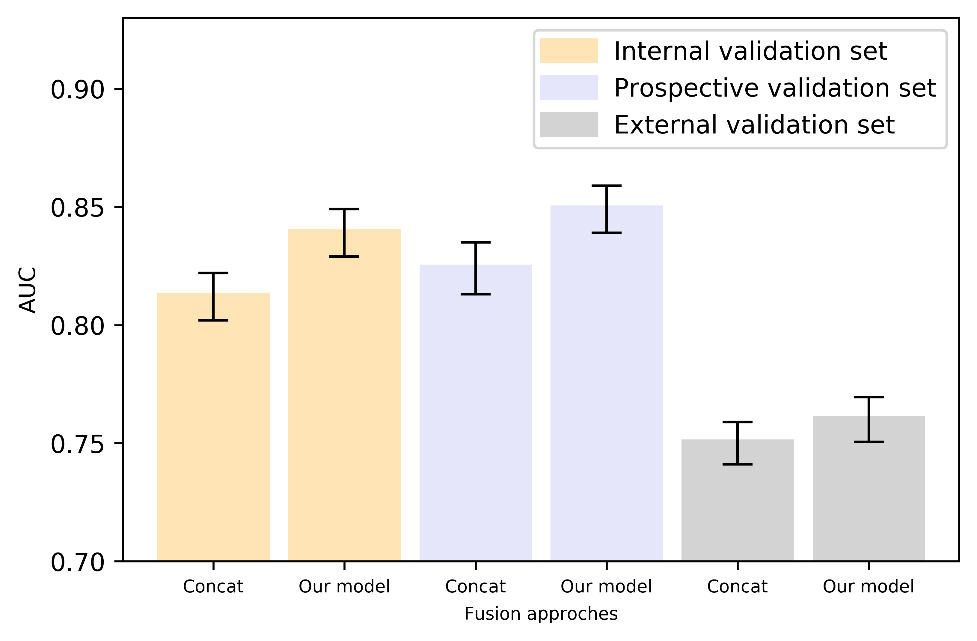


**Figure S3. Comparison with the approach of concatenating all features directly.**

**
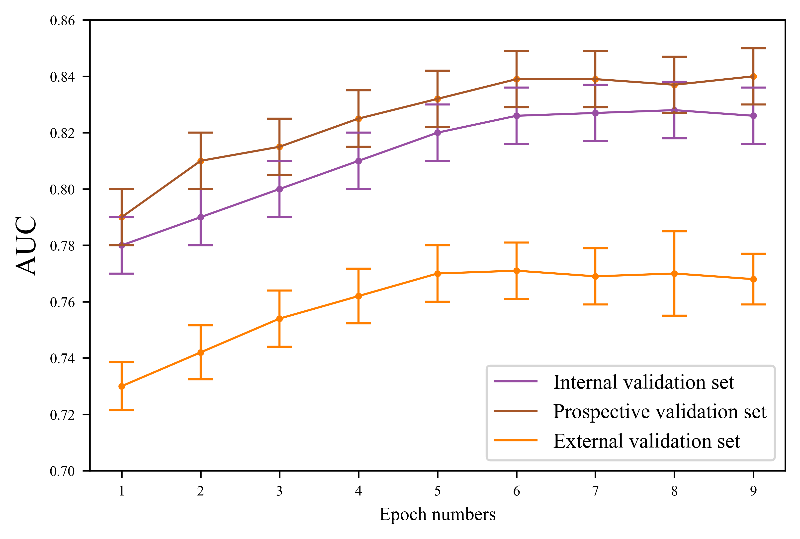
**

**Figure S4. Comparison with different epoch numbers**

**
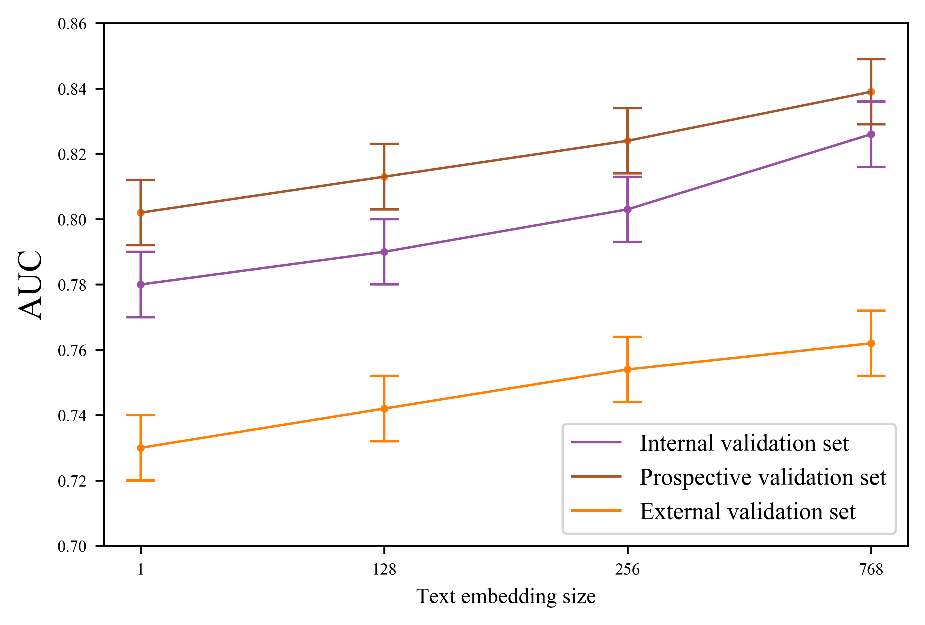
**

**Figure S5. Comparison with different reduction dimension of text feature**

**
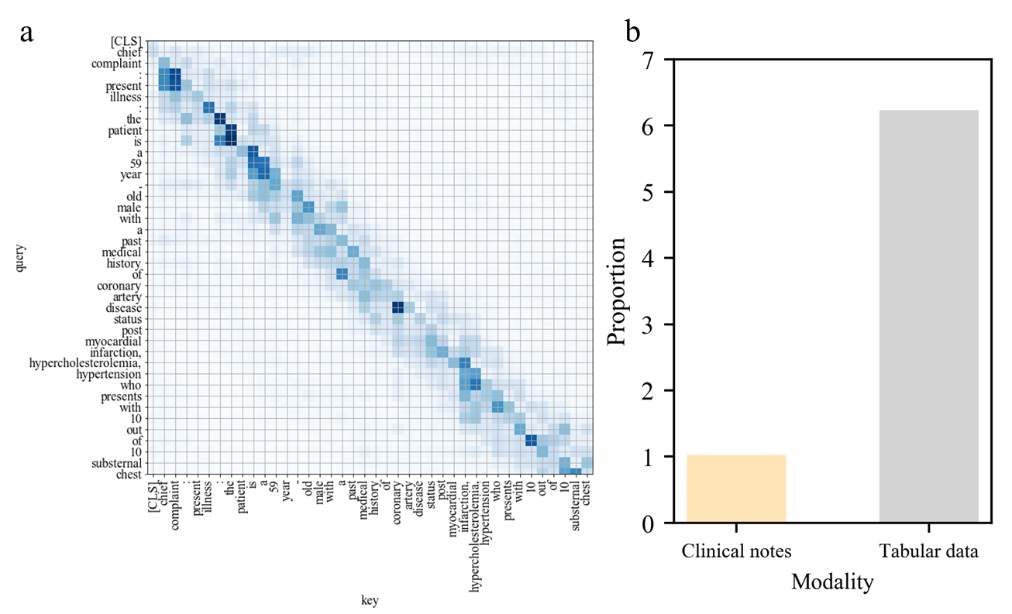
**

**Figure S6. Interpretable predictions of an example**

**Table S1. Names of clinical variables**

| Type (number) | Feature name  (n=52) |
| --- | --- |
| Basic information (9) | Age, Gender, Weight, BMI, CCI score, Days before ICU admission, Ethnicity, Admission type, Elective surgery |
| Vital signs (8) | SBP(mean), MBP(mean), Respiration rate(mean), Temperature(mean), Heart rate(mean), GCS(min), SpO2(min), FiO2(max), |
| Laboratory tests (31) | Creatinine(max), Bilirubin(max), Platelet(min), BUN(max), White blood cell(max), Glucose(max), Hematocrit(max), Potassium(max), Sodium(max), Bicarbonate(max), Bicarbonate(min), Albumin(min), ALT(max), AST(max), ALP(max), PT(max), PTT(max), INR(min), Hemoglobin(min), Chloride(min), Lactate(max), Base Excess(min), Troponin(max), Lymphocytes(max), Lymphocytes(min), Neutrophils(min), Magnesium(max), Anion gap(max), PaO2(min), PaCO2(max), PaO2/FiO2 |
| Outputs (1) | Urine outputs |
| Treatments (1) | Mechanical ventilation |
| Physical frailty (2) | Activity, Fall risk |
| ***Abbreviations****: BMI, body mass index; CCI, Charlson comorbidity index; SBP, systolic blood pressure; MBP, mean blood pressure; GCS, Glasgow coma score; SpO2, oxygen saturation; FiO2, fraction of inspired oxygen; PaO2,* *partial pressure of oxygen; PaCO2, pressure of carbon dioxide; SpO2/FiO2, oxygenation index; BUN, blood urea nitrogen; ALT, alanine aminotransferase; AST, aspartate transaminase; ALP, alkaline phosphatase; PT, prothrombin time; PTT, partial thromboplastin time; INR, international normalized ratio;* | |

**Table S2. The missing ratio of all variables in four study cohorts**

|  | **Development set (%)** | **Internal validation set (%)** | **Prospective validation set (%)** | **External validation set (%)** |
| --- | --- | --- | --- | --- |
| **Demographics** | | | | |
| Age | 0 | 0 | 0 | 0 |
| Gender | 0 | 0 | 0 | 0 |
| Weight | 3.354 | 3.765 | 0.844 | 1.964 |
| BMI | 33.547 | 34.762 | 36.181 | 2.557 |
| Elective surgery | 0 | 0 | 0 | 3.445 |
| CCI score | 0 | 0 | 0 | 0.713 |
| Length of stay before ICU | 0 | 0 | 0 | 0 |
| Admission type | 0 | 0 | 0 | 0 |
| Ethnicity | 0 | 0 | 0 | 0 |
| **Treatments** | | | | |
| Mechanical ventilation | 0 | 0 | 0 | 0 |
| **Laboratory tests** | | | | |
| Creatinine (max) | 0.1 | 0.08 | 0.264 | 0.996 |
| Bilirubin (max) | 49.194 | 51.141 | 34.757 | 25.283 |
| Platelet (min) | 0.36 | 0.32 | 0.316 | 2.691 |
| BUN (max) | 0.12 | 0.2 | 0.264 | 1.144 |
| White blood cell (max) | 0.42 | 0.4 | 0.369 | 1.601 |
| Glucose (max) | 0.27 | 0.32 | 0.791 | 1.736 |
| Hematocrit (max) | 0.16 | 0.2 | 0.316 | 1.332 |
| Potassium (max) | 0.15 | 0.12 | 0.422 | 1.628 |
| Sodium (max) | 0.13 | 0.16 | 0.316 | 1.076 |
| Bicarbonate (min) | 0.32 | 0.36 | 0.264 | 5.853 |
| Bicarbonate (max) | 0.32 | 0.36 | 0.264 | 5.853 |
| PaO2/FiO2 | 65.812 | 65.799 | 74.209 | 72.914 |
| Albumin (min) | 59.926 | 61.794 | 52.901 | 21.757 |
| ALT (max) | 48.784 | 50.421 | 34.757 | 39.397 |
| AST (max) | 48.744 | 50.18 | 34.019 | 38.765 |
| ALP (max) | 49.625 | 51.221 | 34.283 | 39.384 |
| PT (max) | 7.088 | 7.209 | 4.483 | 29.508 |
| PTT (max) | 7.228 | 7.609 | 4.747 | 47.188 |
| INR (min) | 7.068 | 7.209 | 4.483 | 28.081 |
| Hemoglobin (min) | 0.27 | 0.28 | 0.316 | 2.274 |
| Chloride (min) | 0.28 | 0.2 | 0.264 | 1.951 |
| PaO2 (min) | 34.738 | 34.962 | 50.949 | 42.371 |
| PaCO2 (max) | 34.738 | 34.962 | 50.949 | 43.582 |
| Lactate (max) | 52.918 | 52.783 | 56.593 | 54.682 |
| Base excess (min) | 34.738 | 34.962 | 50.949 | 53.687 |
| Troponin (max) | 56.132 | 57.99 | 59.968 | 92.573 |
| Lymphocytes (max) | 54.11 | 55.266 | 49.578 | 31.929 |
| Lymphocytes (min) | 54.11 | 55.266 | 49.578 | 31.929 |
| Neutrophils (min) | 53.859 | 55.106 | 49.525 | 36.814 |
| Magnesium (max) | 3.163 | 3.484 | 2.162 | 33.073 |
| Anion gap (max) | 1.492 | 1.642 | 0.264 | 24.664 |
| **Urine output** | | | | |
| Urine output | 2.383 | 2.443 | 3.059 | 26.211 |
| **Vital signs** | | | | |
| GCS (min) | 0 | 0 | 0 | 27.651 |
| Heart rate (mean) | 0 | 0 | 0 | 8.867 |
| MBP (mean) | 0 | 0 | 0 | 0.148 |
| SBP (mean) | 0 | 0 | 0 | 0.188 |
| Respiratory rate (mean) | 0 | 0 | 0 | 9.365 |
| Temperature (mean) | 0 | 0 | 0 | 3.996 |
| SpO2 (min) | 0 | 0 | 0 | 21.058 |
| FiO2 (max) | 52.388 | 53.825 | 62.869 | 46.717 |
| **Physical frailty** | | | | |
| Activity | 0.4 | 0.681 | 0.158 | 36.249 |
| Fall risk | 0 | 0 | 0 | 0 |

**Table S2. Detailed performance of the model with different text types of notes**

| **Data set** | **Individual text** | AUROC | F1 score | AUPRC |
| --- | --- | --- | --- | --- |
| **Internal validation set** | **Chief complaint** | 0.790 (0.780-0.801) | 0.417 (0.400-0.434) | 0.252 (0.239-0.265) |
|  | **History of present illness** | 0.791 (0.781-0.803) | 0.404 (0.385-0.418) | 0.241 (0.227-0.254) |
|  | **Medical history** | 0.809 (0.797-0.818) | 0.437 (0.414-0.456) | 0.266 (0.249-0.282) |
|  | **Admission medication** | 0.789 (0.779-0.800) | 0.409 (0.392-0.426) | 0.245 (0.232-0.259) |
|  | **Physical exam** | 0.797 (0.788-0.807) | 0.420 (0.390-0.441) | 0.254 (0.234-0.268) |
| **Prospective validation set** | **Chief complaint** | 0.835 (0.829-0.845) | 0.485 (0.473-0.499) | 0.308 (0.297-0.321) |
|  | **History of present illness** | 0.840 (0.831-0.846) | 0.482 (0.446-0.503) | 0.306 (0.279-0.323) |
|  | **Medical history** | 0.844 (0.838-0.849) | 0.534 (0.518-0.554) | 0.352 (0.337-0.367) |
|  | **Admission medication** | 0.834 (0.826-0.842) | 0.499 (0.477-0.533) | 0.321 (0.302-0.349) |
|  | **Physical exam** | 0.841 (0.834-0.850) | 0.496 (0.475-0.523) | 0.317 (0.299-0.339) |
| **External test set** | **History of present illness** | 0.759 (0.754-0.764) | 0.399 (0.390-0.408) | 0.241 (0.234-0.247) |
|  | **Medical history** | 0.759 (0.754-0.763) | 0.407 (0.400-0.415) | 0.246 (0.240-0.252) |
|  | **Admission medication** | 0.757 (0.752-0.762) | 0.401 (0.393-0.411) | 0.242 (0.235-0.248) |
|  | **Physical exam** | 0.762 (0.757-0.767) | 0.396 (0.377-0.410) | 0.238 (0.226-0.248) |

**Table S4. The characteristic of the development set**

|  | **Overall**  **(n=9,989)** | **Survivor**  **(n=8,585)** | **Non-survivor**  **(n=1,404)** | **P-Value** |
| --- | --- | --- | --- | --- |
| **Age, median [Q1,Q3]** | 75 [65,84] | 74 [64,83] | 79 [70,86] | <0.001 |
| **Female, n (%)** | 4637 (46.4) | 3952 (46.0) | 685 (48.8) | 0.059 |
| **Admission type, n (%)** |  |  |  | <0.001 |
| **ELECTIVE** | 754 (7.5) | 719 (8.4) | 35 (2.5) |  |
| **EMERGENCY** | 9235 (92.5) | 7866 (91.6) | 1369 (97.5) |  |
| **Ethnicity, n (%)** |  |  |  | <0.001 |
| **Asian** | 202 (2.0) | 178 (2.1) | 24 (1.7) |  |
| **Black** | 860 (8.6) | 771 (9.0) | 89 (6.3) |  |
| **Hispanic** | 239 (2.4) | 207 (2.4) | 32 (2.3) |  |
| **Other** | 1592 (15.9) | 1287 (15.0) | 305 (21.7) |  |
| **White** | 7096 (71.0) | 6142 (71.5) | 954 (67.9) |  |
| **Weight, median [Q1,Q3]** | 78.6 [65.6,94.4] | 79.5 [66.2,95.1] | 74.5 [61.4,88.5] | <0.001 |
| **BMI, median [Q1,Q3]** | 27.7 [24.1,32.6] | 27.9 [24.2,32.9] | 26.2 [22.8,31.3] | <0.001 |
| **Elective surgery, n (%)** | 587 (5.9) | 562 (6.5) | 25 (1.8) | <0.001 |
| **Mechanical ventilation, n (%)** | 4160 (41.6) | 3409 (39.7) | 751 (53.5) | <0.001 |
| **Activity, n (%)** |  |  |  | <0.001 |
| **Bed** | 7347 (73.8) | 6095 (71.2) | 1252 (89.8) |  |
| **Sit** | 1759 (17.7) | 1657 (19.4) | 102 (7.3) |  |
| **Stand** | 843 (8.5) | 803 (9.4) | 40 (2.9) |  |
| **Fall risk, n (%)** | 3334 (33.4) | 2816 (32.8) | 518 (36.9) | 0.003 |
| **CCI score, median [Q1,Q3]** | 7.0 [5.0,8.0] | 7.0 [5.0,8.0] | 7.0 [6.0,9.0] | <0.001 |
| **Length of stay before ICU, median [Q1,Q3]** | 0.1 [0.0,1.1] | 0.1 [0.0,1.0] | 0.1 [0.0,1.3] | 0.125 |
| **Length of ICU durations, median [Q1,Q3]** | 3.0 [1.8,5.5] | 2.9 [1.8,5.0] | 4.7 [2.4,9.0] | <0.001 |
| **Length of hospital durations, median [Q1,Q3]** | 8.9 [5.7,14.6] | 8.9 [5.8,14.5] | 8.6 [4.5,15.6] | 0.001 |

**Table S5. The characteristic of the internal validation set**

|  | **Overall**  **(n=2,497)** | **Survivor**  **(n=2,146)** | **Non-survivor**  **(n=351)** | **P-Value** |
| --- | --- | --- | --- | --- |
| **Age, median [Q1,Q3]** | 75 [64,83] | 74 [64,83] | 79 [68,86] | <0.001 |
| **Female, n (%)** | 1151 (46.1) | 996 (46.4) | 155 (44.2) | 0.467 |
| **Admission type, n (%)** |  |  |  | 0.004 |
| **ELECTIVE** | 198 (7.9) | 184 (8.6) | 14 (4.0) |  |
| **EMERGENCY** | 2299 (92.1) | 1962 (91.4) | 337 (96.0) |  |
| **Ethnicity, n (%)** |  |  |  | 0.016 |
| **Asian** | 47 (1.9) | 38 (1.8) | 9 (2.6) |  |
| **Black** | 230 (9.2) | 211 (9.8) | 19 (5.4) |  |
| **Hispanic** | 53 (2.1) | 50 (2.3) | 3 (0.9) |  |
| **Other** | 377 (15.1) | 316 (14.7) | 61 (17.4) |  |
| **White** | 1790 (71.7) | 1531 (71.3) | 259 (73.8) |  |
| **Weight, median [Q1,Q3]** | 78.0 [65.4,94.0] | 79.0 [65.8,95.0] | 74.5 [62.7,88.8] | <0.001 |
| **BMI, median [Q1,Q3]** | 27.5 [23.8,32.6] | 27.7 [23.8,32.7] | 26.9 [23.6,31.3] | 0.133 |
| **Elective surgery, n (%)** | 166 (6.6) | 156 (7.3) | 10 (2.8) | 0.003 |
| **Mechanical ventilation, n (%)** | 1030 (41.2) | 848 (39.5) | 182 (51.9) | <0.001 |
| **Activity, n (%)** |  |  |  | <0.001 |
| **Bed** | 1801 (72.6) | 1497 (70.2) | 304 (87.1) |  |
| **Sit** | 444 (17.9) | 411 (19.3) | 33 (9.5) |  |
| **Stand** | 235 (9.5) | 223 (10.5) | 12 (3.4) |  |
| **Fall risk, n (%)** | 836 (33.5) | 706 (32.9) | 130 (37.0) | 0.144 |
| **CCI score, median [Q1,Q3]** | 7.0 [5.0,9.0] | 7.0 [5.0,8.0] | 8.0 [6.0,10.0] | <0.001 |
| **Length of stay before ICU, median [Q1,Q3]** | 0.1 [0.0,1.3] | 0.1 [0.0,1.2] | 0.1 [0.0,1.9] | 0.212 |
| **Length of ICU durations, median [Q1,Q3]** | 3.0 [1.8,5.5] | 2.9 [1.8,5.0] | 5.0 [2.5,9.7] | <0.001 |
| **Length of hospital durations, median [Q1,Q3]** | 9.2 [5.8,14.7] | 9.0 [5.8,14.2] | 10.0 [5.3,18.2] | 0.143 |

**Table S6. The characteristic of the prospective validation set**

|  | **Overall**  **(n=1,896)** | **Survivor**  **(n=1,603)** | **Non-survivor**  **(n=293)** | **P-Value** |
| --- | --- | --- | --- | --- |
| **Age, median [Q1,Q3]** | 74 [64,82] | 73 [63,81] | 78 [70,86] | <0.001 |
| **Female, n (%)** | 786 (41.5) | 667 (41.6) | 119 (40.6) | 0.8 |
| **Admission type, n (%)** |  |  |  | 0.033 |
| **ELECTIVE** | 70 (3.7) | 66 (4.1) | 4 (1.4) |  |
| **EMERGENCY** | 1826 (96.3) | 1537 (95.9) | 289 (98.6) |  |
| **Ethnicity, n (%)** |  |  |  | 0.004 |
| **Asian** | 38 (2.0) | 31 (1.9) | 7 (2.4) |  |
| **Black** | 117 (6.2) | 106 (6.6) | 11 (3.8) |  |
| **Hispanic** | 40 (2.1) | 35 (2.2) | 5 (1.7) |  |
| **Other** | 516 (27.2) | 411 (25.6) | 105 (35.8) |  |
| **White** | 1185 (62.5) | 1020 (63.6) | 165 (56.3) |  |
| **Weight, median [Q1,Q3]** | 79.8 [66.5,97.2] | 80.2 [66.4,97.7] | 77.7 [66.9,93.6] | 0.142 |
| **BMI, median [Q1,Q3]** | 28.8 [24.4,33.8] | 28.8 [24.5,34.0] | 28.4 [23.4,32.8] | 0.137 |
| **Elective surgery, n (%)** | 50 (2.6) | 48 (3.0) | 2 (0.7) | 0.038 |
| **Mechanical ventilation, n (%)** | 720 (38.0) | 562 (35.1) | 158 (53.9) | <0.001 |
| **Activity, n (%)** |  |  |  | <0.001 |
| **Bed** | 1064 (56.2) | 835 (52.2) | 229 (78.2) |  |
| **Sit** | 441 (23.3) | 404 (25.2) | 37 (12.6) |  |
| **Stand** | 388 (20.5) | 361 (22.6) | 27 (9.2) |  |
| **Fall risk, n (%)** | 1896 (100.0) | 1603 (100.0) | 293 (100.0) | 1 |
| **CCI score, median [Q1,Q3]** | 7.0 [6.0,9.0] | 7.0 [5.0,9.0] | 8.0 [7.0,10.0] | <0.001 |
| **Length of stay before ICU, median [Q1,Q3]** | 0.1 [0.0,0.9] | 0.1 [0.0,0.9] | 0.1 [0.0,0.6] | 0.002 |
| **Length of ICU durations, median [Q1,Q3]** | 2.9 [1.7,5.3] | 2.8 [1.7,5.0] | 4.5 [2.5,8.1] | <0.001 |
| **Length of hospital durations, median [Q1,Q3]** | 9.8 [6.0,15.7] | 9.8 [6.3,15.8] | 8.0 [3.8,14.8] | <0.001 |

**Table S7. The characteristic of the external validation set**

|  | **Overall**  **(n=7,432)** | **Survivor**  **(n=6,317)** | **Non-survivor**  **(n=1,115)** | **P-Value** |
| --- | --- | --- | --- | --- |
| **Age, median [Q1,Q3]** | 73 [62,82] | 72 [62,82] | 77 [66,84] | <0.001 |
| **Female, n (%)** | 3470 (46.7) | 2951 (46.7) | 519 (46.5) | 0.943 |
| **Admission type, n (%)** |  |  |  | 0.551 |
| **ELECTIVE** | 1856 (25.0) | 1586 (25.1) | 270 (24.2) |  |
| **EMERGENCY** | 5576 (75.0) | 4731 (74.9) | 845 (75.8) |  |
| **Ethnicity, n (%)** |  |  |  | 0.002 |
| **Asian** | 140 (1.9) | 119 (1.9) | 21 (1.9) |  |
| **Black** | 938 (12.6) | 830 (13.1) | 108 (9.7) |  |
| **Hispanic** | 370 (5.0) | 298 (4.7) | 72 (6.5) |  |
| **Other** | 407 (5.5) | 354 (5.6) | 53 (4.8) |  |
| **White** | 5577 (75.0) | 4716 (74.7) | 861 (77.2) |  |
| **Weight, median [Q1,Q3]** | 82.8 [68.0,102.0] | 83.4 [68.4,102.3] | 79.4 [65.6,98.5] | <0.001 |
| **BMI, median [Q1,Q3]** | 28.9 [24.4,35.0] | 29.1 [24.5,35.1] | 27.8 [23.6,33.8] | <0.001 |
| **Elective surgery, n (%)** | 6974 (97.2) | 5895 (97.0) | 1079 (98.4) | 0.008 |
| **Mechanical ventilation, n (%)** | 3731 (50.2) | 2981 (47.2) | 750 (67.3) | <0.001 |
| **Activity, n (%)** |  |  |  | <0.001 |
| **Bed** | 3955 (83.5) | 3309 (82.2) | 646 (90.7) | <0.001 |
| **Sit** | 279 (5.9) | 257 (6.4) | 22 (3.1) |  |
| **Stand** | 504 (10.6) | 460 (11.4) | 44 (6.2) |  |
| **Fall risk, n (%)** | 1374 (18.5) | 1177 (18.6) | 197 (17.7) | 0.47 |
| **CCI score, median [Q1,Q3]** | 5.0 [4.0,6.0] | 5.0 [4.0,6.0] | 5.0 [5.0,7.0] | <0.001 |
| **Length of stay before ICU, median [Q1,Q3]** | 0.2 [0.1,0.8] | 0.2 [0.1,0.7] | 0.2 [0.1,1.3] | <0.001 |
| **Length of ICU durations, median [Q1,Q3]** | 2.8 [1.8,4.9] | 2.7 [1.8,4.6] | 3.9 [2.1,7.1] | <0.001 |
| **Length of hospital durations, median [Q1,Q3]** | 7.2 [4.3,11.9] | 7.2 [4.5,11.8] | 6.9 [3.7,13.0] | 0.016 |

**Table S8. Detailed performance of multimodal model vs unimodal models**

| **Data set** | **Modality** | **Prediction model** | **AUROC** | **F1 score** | **AUPRC** |
| --- | --- | --- | --- | --- | --- |
| **Internal validation set** | **Text and tabular** | Multimodal Attention (Ours) | 0.839 (0.827-0.852) | 0.469 (0.430-0.510) | 0.293 (0.264-0.321) |
|  | **Tabular only** | Random Forest | 0.828 (0.818-0.840) | 0.454 (0.417-0.478) | 0.278 (0.252-0.298) |
|  |  | Logistic regression | 0.824 (0.814-0.836) | 0.426 (0.390-0.477) | 0.259 (0.237-0.291) |
|  |  | Fully connected Neural Network | 0.792 (0.780-0.803) | 0.404 (0.347-0.441) | 0.241 (0.205-0.266) |
|  | **Text only** | Clinical Bert | 0.701 (0.689-0.717) | 0.330 (0.312-0.345) | 0.193 (0.180-0.203) |
| **prospective validation set** | **Text and tabular** | Multimodal Attention (Ours) | 0.849 (0.841-0.857) | 0.488 (0.475-0.501) | 0.312 (0.301-0.324) |
|  | **Tabular only** | Random Forest | 0.839 (0.833-0.845) | 0.482 (0.459-0.533) | 0.310 (0.292-0.349) |
|  |  | Logistic regression | 0.843 (0.839-0.848) | 0.483 (0.460-0.511) | 0.310 (0.293-0.331) |
|  |  | Fully connected Neural Network | 0.796 (0.790-0.802) | 0.438 (0.428-0.446) | 0.272 (0.263-0.278) |
|  | **Text only** | Clinical Bert | 0.686 (0.676-0.698) | 0.346 (0.325-0.368) | 0.207 (0.194-0.222) |
| **External validation set** | **Text and tabular** | Multimodal Attention (Ours) | 0.767 (0.762-0.772) | 0.415 (0.408-0.421) | 0.252 (0.246-0.256) |
|  | **Tabular only** | Random Forest | 0.755 (0.748-0.761) | 0.413 (0.403-0.424) | 0.249 (0.242-0.258) |
|  |  | Logistic regression | 0.752 (0.746-0.759) | 0.426 (0.418-0.434) | 0.257 (0.251-0.264) |
|  |  | Fully connected Neural Network | 0.751 (0.745-0.757) | 0.394 (0.356-0.407) | 0.236 (0.213-0.245) |
|  | **Text only** | Clinical Bert | 0.605 (0.598-0.611) | 0.291 (0.284-0.296) | 0.176 (0.172-0.180) |

**Table S9. Performance comparisons of the pretrained Bert model**

| Data set | Pretrained Bert model | AUROC |
| --- | --- | --- |
| Internal validation set | Bert base | 0.525 |
|  | BioRoBerta | 0.528 |
|  | BioBert | 0.582 |
|  | Clinical Bert | 0.701 |
| Prospective validation set | Bert base | 0.526 |
|  | BioRoBerta | 0.558 |
|  | BioBert | 0.584 |
|  | Clinical Bert | 0.686 |
| External validation set | Bert base | 0.521 |
|  | BioRoBerta | 0.455 |
|  | BioBert | 0.572 |
|  | Clinical Bert | 0.605 |
